# A Case of Haemorrhagic Constrictive Pericarditis with Bilateral Pleural Effusions

**DOI:** 10.1155/2016/8142134

**Published:** 2016-10-11

**Authors:** Hans A. Reyes, Julie Islam, Soheila Talebi, Eder Cativo, Savi Mushiyev, Gerald Pekler, Ferdinand Visco

**Affiliations:** ^1^Department of Medicine, New York Medical College, Metropolitan Hospital Center, Valhalla, NY, USA; ^2^Division of Cardiology, New York Medical College, Metropolitan Hospital Center, Valhalla, NY, USA

## Abstract

Presentation of pericardial disease is diverse, with the viral aetiology being the most common cause; however, when haemorrhagic pericardial effusion is present, these causes are narrowed to few aetiologies. We present a case of a young female of African descent who presented with diffuse abdominal pain and vomiting. Initial work-up showed pericardial effusion with impending echocardiographic findings of cardiac tamponade and bilateral pleural effusions. Procedures included a left video-assisted thoracoscopic surgery (VATS) with pericardial window. We consider that it is important for all physicians to be aware of not only typical presentation but also atypical and unusual clinical picture of pericardial disease.

## 1. Introduction

For a haemorrhagic pericardial effusion, the culprits are limited to a few aetiologies including myocardial infarction, trauma, tuberculosis, malignancy, or aortic dissection, all of which can lead to cardiac tamponade, hemodynamic instability, and compromise. Moreover, in young patients without remarkable history of cardiac disease, infections should be ruled out first.

## 2. Case

**Figure 1:**
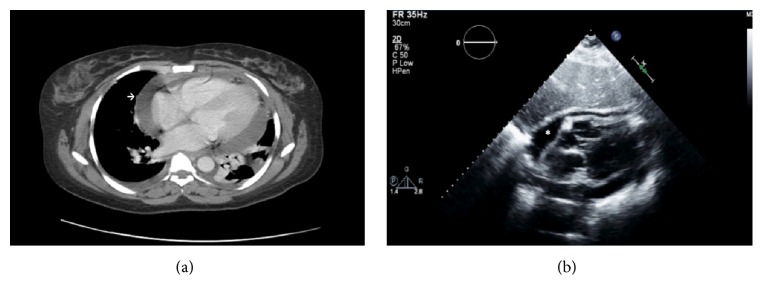
(a) CT showed cardiomegaly with large pericardial effusion (arrow). Bilateral pleural effusions. Left lower lobe is nearly completely collapsed. (b) Parasternal long-axis echocardiogram showed pericardial effusion of moderate size with diastolic compression of the right atrium (asterisk).

A 31-year-old female from Mali who had been living in the United States for the past three years presented to the Emergency Department complaining of diffuse abdominal pain for 6 days and one episode of nonbilious vomiting. Past medical history included uncontrolled hypertension due to medication nonadherence. Physical examination revealed blood pressure of 161/109 mmHg, heart rate of 114 bpm, respiratory rate of 18 per minute, O_2_sat of 98% on room air, and temperature of 37°C. There were normal heart sounds and normal lung sounds, there were no rubs and no JVD, and abdomen was soft with RUQ tenderness and no peripheral edema. An EKG confirmed sinus tachycardia with left axis deviation, normal voltage, ST segment, and QRS complexes. Laboratory data were as follows: WBC of 8020/*µ*L, Hb/Hcto of 10/31, normal electrolytes and renal function, hepatic function remarkable for hypoalbuminemia, 2.6 g/dL, and normal coagulation profile. Cardiomegaly and blunting of the right and left costophrenic angles were evident on chest X-ray. An abdominal and pelvis CT was ordered due to persistence of abdominal pain despite opioids analgesics, and it incidentally showed a large pericardial effusion along with bilateral pleural effusions (Figure 1(a)), worse on the left. No underlying aetiology for her abdominal pain could be visualized on abdominal CT.

Shortly thereafter, the patient started to complain of shortness of breath, chest tightness, and orthopnoea, which varied with position and was relieved by sitting upright and leaning forward. A 2D echocardiogram showed preserved LVEF, >55%, pericardial effusion of moderate size with right atrial late diastolic collapse, dilated nonpulsatile inferior vena cava (IVC), mitral flow velocity paradoxus (≥25% change in E wave flow velocity during inspiration compared to expiration), and A′ wave greater than E′ wave in tissue Doppler (consistent with impaired filling pattern, which can be restrictive or constrictive) (Figure 1(b)), and she was immediately transferred to Coronary Care Unit. Pulsus paradoxus was not ever greater than 10 mmHg during her stay; no fever was reported as well. An IR-guided pericardiocentesis was performed showing 200 mL of blood-tinged fluid, RBC of 508,500/mm^3^, and WBC of 950/mm^3^ with 88% segments. Cytology study was negative for malignant cells. In the repeated 2D echocardiogram after drainage, E′ wave became greater than A′ wave in tissue Doppler, which is more consistent with pericardial constriction. Laboratory work-up for autoimmune aetiologies, cardiotropic viruses, and bacterial infections including tuberculosis were unremarkable for any positive findings. The rest of the ancillary laboratory revealed iron deficiency anaemia and elevation of ESR and C-reactive protein, 50 mm/h and 251 mg/L, respectively. HIV test, PPD, and cancer markers such as CEA and CA 19-9 were also negative. Then, initial subxiphoid pericardial window attempt was unsuccessful due to a thickened pericardium firmly attached to the heart and sternum. The following day, left VATS with pericardial window was done. Again, the pericardium appeared to be thickened and was extremely adherent to the myocardium. Fluid from pericardial and pleural effusions was again sanguineous with no malignant cells. Cultures remained negative; adenosine deaminase (ADA) from pleural fluid and PCR assay for tuberculosis from pericardial fluid came back normal as well. Biopsy of pericardial tissue was consistent with chronic pericarditis, while pleural biopsy confirmed reactive pleuritis. Acid fast and silver stains were normal.

**Figure 2:**
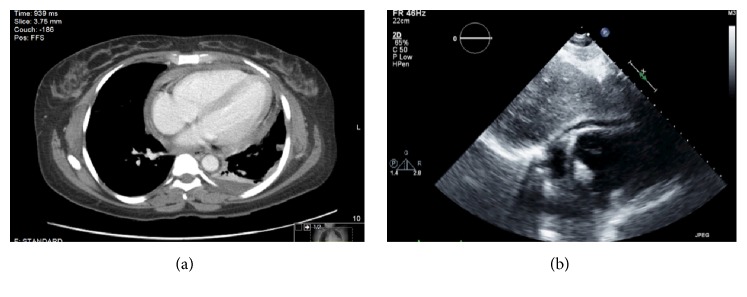
(a) Small pericardial effusion. Subsegmental atelectasis at the left lower lobe. Small left pleural effusion; previous right pleural effusion has resolved. (b) Parasternal long-axis echocardiogram showed small pericardial effusion with no evidence of echo cardiac tamponade.

After extensive negative work-up, patient was started on colchicine 0.6 mg daily; she progressively and slowly improved during the rest of her stay. Repeated chest CT and echocardiogram after almost one month of hospitalization showed only small accumulation of pericardial fluid (Figures 2(a) and 2(b)).

Upon discharge, the patient was seen in the medicine and cardiology clinic as outpatient at one-month and two-month follow-up. She was doing well and was asymptomatic and adherent to medication including colchicine 0.6 mg daily.

## 3. Discussion

We are describing this case of idiopathic haemorrhagic constrictive pericarditis presenting with impending echocardiographic findings of cardiac tamponade. When a patient presents with a haemorrhagic pericardial effusion, it is always a challenge to determine the aetiology; however, tuberculosis and malignancy should be ruled out first [1–3]. Our patient had emigrated from Mali three years prior to presentation, provoking high suspicion of tuberculosis; nevertheless, repeated AFBs from sputum and fluids, cultures, PPD, and ADA from pleural fluid and PCR from pericardial fluid came back negative. Viral, fungal infection and autoimmune diseases were ruled out as well. We thoroughly evaluated secondary causes for haemorrhagic effusion; however, ancillary tests were not diagnostic. As aforementioned, haemorrhagic effusion can also be seen in patients with pericarditis after myocardial infarction, trauma, aortic dissection, or uremic pericarditis but this was not our patient's presentation. Roughly 80 to 90% of pericardial effusions will remain idiopathic after an extensive work-up [1, 2].

Our patient's initial presentation was not typical for pericardial tamponade. However, one may argue that, in subacute or chronic processes, typical signs like tachycardia, hypotension, JVD, and diminished heart sounds have low sensitivity. Even pulsus paradoxus, the cornerstone in cardiac tamponade, has a variable incidence ranging from 12% to 75% [3]. Echocardiography remains an important diagnostic tool which allows us to identify signs of tamponade such as collapse of the right atrium, engorgement of the IVC, or right ventricular diastolic collapse. It is, however, important to note that not all effusions will need surgical intervention. It should only be considered in patients with hemodynamic instability or if purulent fluid was noted during an intervention [4, 5].

The fact that the pericardium was thickened and extremely adherent to myocardium during the two surgical interventions along with constrictive filling pattern on echocardiogram and biopsy findings support a chronic process in this patient. This clinical picture is consistent with constrictive pericarditis. It is a rare type of pericarditis which develops insidiously and in many cases no aetiology is ever determined. The principal causes are idiopathic and cardiothoracic surgery and previous radiation [6]. Our patient did not have a previous history of surgery or radiation; again infections, uraemia, neoplasms, connective tissue disorders, and trauma were ruled out. Constrictive pericarditis is a potentially curable disease if it is diagnosed early but could be potentially fatal if it was overlooked. The reason why the patient developed bilateral pleural effusions is still unknown; however, the initial pericardial effusion and quantity of inflammation with fibrin accumulation may have subsequently triggered pleural inflammation and effusion by proximity.

For cases where aetiology remains unclear as in our patient, studies recommend the use of colchicine and NSAIDs [1, 7]. Aside from this atypical presentation, the slow but progressive response to colchicine leads us to believe in an idiopathic aetiology or it could be related to previously unrecognized viral pericarditis. We decided not to use NSAIDs due to the concern that this may worsen the haemorrhagic effusion and delay recovery.

In short, no matter how atypical the clinical picture may be, the majority of cases of pericarditis are of unknown aetiology. At her two-month follow-up, the patient has remained asymptomatic and has responded well to colchicine alone. An idiopathic cause should be considered only after all other possibilities have been exhausted, even if a large haemorrhagic effusion is seen. We consider that the patient might be at risk of developing long-term constrictive pericarditis which is associated with high morbidity. Additional echocardiograms will be beneficial for her during her subsequent follow-ups.
